# Supplementary material for: Changes in Inflammatory Cytokines in Responders and Non-Responders to TNFα Inhibitor and IL-17A Inhibitor: A Study Examining Psoriatic Arthritis Patients
Source: Int J Mol Sci. 2024 Mar 5;25(5):3002. doi: 10.3390/ijms25053002 (PMC10932211; doi:10.3390/ijms25053002)
Supplement: Supplementary file 1 [file ijms-25-03002-s001.zip › Table S1.pdf]

Table S1: Biomarker levels at baseline in PsA patients initiating either TNFi, IL-17Ai or MTX

	All (n = 67)	n	TNFi (n = 28)	n	IL-17Ai (n =19)	n	MTX (n =20)	n	p-value
VCAM-1	8.350x10^{6} (6.374x10^{6}-9.931x10^{6})	64	7.325x10^{6} (6.601x10^{6}-9.934x10^{6})	27	8.613x10^{6} (6.909x10^{6}-10.195x10^{6})	18	9.152 x10^{6} (7.588 x10^{6}-9.839 x10^{6})	19	p = 0.486
SAA	5.968x10^{7} (2.951x10^{7}-6.063x10^{8})	55	4.254x10^{7} (2.568x10^{7}-7.844x10^{7})	23	6.255x10^{7} (3.549x10^{7}-3.302x10^{8})	15	8.555x10^{7} (4.728x10^{7}-1.706x10^{8})	16	p = 0.115
ICAM-1	7.900x10^{6} (6.477x10^{6}-9.667x10^{6})	60	7.857x10^{6} (6.349x10^{6}-8.995x10^{6})	25	8.831x10^{6} (7.249x10^{6}-9.895x10^{6})	18	7.304x10^{6} (6.263x10^{6}-9.691x10^{6})	17	p = 0.660
CRP	9.916x10^{7} (3.910x10^{7}-2.256x10^{8})	60	6.967x10^{7} (2.536x10^{7}-2.046x10^{8})	26	1.600x10^{8} (5.280x10^{7}-2.377x10^{8})	16	8.580 x10^{7} (2.078 x10^{7}-2.056 x10^{7})	17	p = 0.243
MIP-3α	8.823 (5.663-13.612)	67	8.603 (5.637-13.151)	28	10.697 (6.706-13.859)	18	8.808 (5.526-15.047)	20	p = 0.585
IL-31	0.243 (0.145-0.688)	36	0.312 (0.165-0.713)	15	0.191 (0.101-0.243)	12	0.704 (0.486-0.975)	9	**p = 0.027**
IL-27	1335.0 (906.7-1833.1)	66	1122.2 (728.5-1694.0)	28	1206.4 (1002.5-1540.5)	18	1621.9 (1120.1-2018.0)	20	p = 0.112
IL-23	19.245 (8.864-165.521)	12	15.727 (10.279-22.819)	22	26.136 (9.424-32.634)	16	28.842 (7.771-40.381)	17	p = 0.839
IL-22	1.302 (0.702-12.532)	64	1.140 (0.692-1.975)	27	1.528 (0.693-2.376)	18	1.222 (0.816-2.833)	19	p = 0.891
IL-21	6.015 (3.500-9.673)	21	5.442 (3.166-11.024)	11	6.015 (5.223-6.105)	3	8.881 (3.397-11.272)	7	p = 0.965
TNF-α	1.463 (1.198-2.122)	67	1.356 (1.154-2.013)	28	2.163 (1.497-3.265)	19	1.325 (1.112-1.507)	20	**p = 0.003**
IL-8	4.636 (2.847-5.886)	66	4.670 (3.767-5.794)	27	5.287 (4.196-8.000)	19	3.076 (2.498-4.845)	20	p = 0.053
IL-6	1.374 (0.778-2.279)	65	1.421 (0.689-2.849)	27	1.379 (1.066-1.961)	19	1.355 (0.750-2.216)	19	p = 0.859
IL-4	8.517x10^{-3} (5.213 x10^{-3}-17.383 x10^{-3})	40	9.448 x10^{-3} (5.308 x10^{-3}-21.711x10^{-3})	15	11.053 x10^{-3} (6.667 x10^{-3}-19.006x10^{-3})	13	7.885 x10^{-3} (3.684 x10^{-3}-10.757 x10^{-3})	12	p = 0.462
IL-2	0.458 (0.218-0.624)	29	0.398 (0.218-0.576)	13	0.568 (0.064-0.717)	9	0.459 (0.275-0.616)	7	p = 0.839
IL-1β	0.080 (0.047-0.245)	20	0.091 (0.066-0.238)	9	0.061 (0.060-0.112)	5	0.088 (0.033-0.343)	6	p = 0.897
IL-13	0.628 (0.277-1.135)	44	0.703 (0.277-1.159)	20	0.566 (0.283-1.249)	10	0.541 (0.382-0.762)	14	p = 0.604
IL-12p70	0.187 (0.105-0.274)	49	0.165 (0.099-0.328)	22	0.190 (0.158-0.227)	15	0.185 (0.083-0.328)	12	p = 0.909
IL-10	0.120 (0.151-0.280)	64	0.191 (0.154-0.267)	27	0.200 (0.147-0.304)	19	0.229 (0.154-0.278)	18	p = 0.945
IFNγ	4.506 (2.801-7.108)	64	5.202 (3.210-11.384)	27	5.009 (3.389-10.519)	18	3.031 (2.246-5.725)	19	p = 0.112
TSLP	3.027x10^{-6} (6.953x10^{-13}; 1.116x10^{-8})	33	3.865x10^{-9} (1.522x10^{-9}; 8.193x10^{-9})	14	7.306x10^{-9} (4.688x10^{-10}; 2.701x10^{-8})	9	1.895x10^{-9} (1.246x10^{-9}; 1.165x10^{-8})		p = 0.949
IL-9	0.383 (0.218-0.751)	30	0.354 (0.210-1.285)	11	0.509 (0.287-0.772)	9	0.389 (0.171-0.0.454)	10	p = 0.667
IL-3	2.862 (1.471-5.051)	14	1.843 (0.858-3.248)	14	3.559 (1.658-5.539)	7	2.896 (2.814-5.539)	3	p = 0.461
IL-1RA	429.1 (243.4-587.3)	67	426.5 (201.4-556.3)	28	486.1 (355.3-904.2)	19	351.9 (246.9-489.2)	20	p = 0.179
IL-17D	15.966 (12.454-23.262)	59	15.804 (11.932-19.566)	24	13.975 (12.179-19423)	16	20.538 (15.140-29.243)	19	p = 0.086
IL-17C	5.151 (3.674-9.130)	59	5.295 (3.814-9.203)	27	4.668 (3.243-9.070)	17	5.736 (4.041-8.574)	15	p = 0.576
IL-17B	4.254 (3.285-6.988)	56	4.071 (2.663-5.793)	24	4.399 (3.923-8.038)	17	3.644 (3.269-6.746)	15	p = 0.226
IL-17A/F	1.530 (0.906-2.619)	20	1.924 (1.054-3.043)	10	1.056 (0.379-4.182)	4	1.283 (1.069-2.137)	6	p = 0.734
TNF-β	0.135 (0.105-0.200)	42	0.153 (0.115-0.203)	17	0.191 (0.121-0.240)	12	0.110 (0.065-0.140)	13	p = 0.059
IL-7	3.661 (2.213-5.553)	65	4.168 (2.570-6.265)	26	4.445 (2.428-7.346)	19	3.068 (1.945-3.948)	20	p = 0.163
IL-5	0.356 (0.226-0.486)	45	0.362 (0.240-0.431)	18	0.355 (0.230-0.501)	12	0.287 (0.202-0.498)	15	p = 0.920
IL-1α	3.546 (2.249-5.842)	60	3.123 (1.846-5.185)	24	3.187 (2.211-5.763)	17	4.759 (2.636-6.312)	19	p = 0.329
IL-17A	2.578 (1.256-4.748)	59	2.490 (1.415; 5.266)	24	4.445 (1.952-5.784)	17	1.648 (0.783; 2.805)	18	**p = 0.022**
IL-16	256.73 (203.86-298.63)	64	253.18 (200.65-290.22)	26	264.25 (109.99-344.06)	18	256.8 (212.9-294.3)	20	p = 0.890
IL-15	2.204 (1.733-2.701)	65	2.405 (1.964-2.710)	26	2.467 (2.017-2.898)	19	1.678 (1.437-2.118)	20	**p = 0.003**
IL-12/IL-23p40	93.772 (63.423-148.409)	66	84.74 (64.66-123.62)	27	129.512 (79.014-205.536)	19	87.47 (56.86-136.88)	20	p = 0.123
GM-CSF	0.109 (0.073-0.149)	20	0.109 (0.089-0.128)	8	0.088 (0.075-0.113)	5	0.133 (0.068-0.177)	7	p = 0.909
TARC	175.15 (104.51-304.21)	67	142.34 (93.00-277.92)	28	192.0 (120.4-307.9)	19	179.61 (97.87-272.35)	20	p = 0.705
MIP-1β	103.24 (69.29-147.24)	67	89.55 (58.04-123.54)	28	95.97 (69.29-141.21)	19	135.76 (101.74-151.69)	20	p = 0.111
MIP-1α	22.205 (13.498-26.500)	51	16.792 (11.614-25.128)	22	24.263 (19.169-26.582)	14	23.970 (18.717-38.082)	15	p = 0.148
MDC	1392.6 (1019.0-2016,1)	67	1287.2 (900.1-1796.7)	28	1698.0 (1093.2-2181.4)	19	1300.4 (1100.5-1743.6)	20	p = 0.411
MCP-4	80.85 (68.71-124.52)	67	76.56 (69.09-124.39)	28	77.25 (68.33-125.29)	19	99.54 (75.45-125.40)	20	p = 0.532
MCP-1	171.23 (99.93-234.36)	67	130.80 (93.62-212.38)	28	198.49 (91.69-268.87)	19	189.03 (142.57-216.82)	20	p = 0.339
IP-10	509.1 (340.2-724.4)	67	445.8 (306.6-644.1)	28	509.1 (374.1-798.1)	19	655.5 (467.5-769.9)	20	p = 0.182
Eotaxin-3	23.418 (17.050-34.368)	45	22.46 (16.88-31.02)	20	26.68 (21.60-39.24)	13	22.280 (12.466-29.051)	9	p = 0.239
Eotaxin	340.5 (223.6-432.6)	67	251.8 (194.7-385.1)	28	327.1 (235.4-443.5)	19	408.8 (314.3-451.8)	20	**p = 0.044**
bFGF	319.432 (130.323-527.229)	65	313.531 (135.580-510.118)	26	319.43 (126.17-512.83)	19	384.19 (129.70-508.57)	20	p = 0.926

VEGF-D	$2.058x10^4$ ($1.586 x10^4$-$2.656 x10^4$)	65	$2.031x10^4$ ($1.562 x10^4$-$2.667 x10^4$)	27	$2.299x10^4$ ($1.711 x10^4$-$2.808x10^4$)	19	$2.004x10^4$ ($1.592x10^4$-$2.406x10^4$)	20	p = 0.749
VEGF-C	$1.607x10^4$ ($0.911x10^4$-$2.474x10^4$)	62	$1.791x10^3$ ($0.916 x10^3$-$2.454 x10^3$)	27	$1.516x10^3$ ($0.997x10^3$-$2.888x10^3$)	18	$1.355x10^3$ ($0.896x10^3$-$1.755x10^3$)	18	p = 0.513
VEGF-A	$2.007x10^4$ ($1.508x10^4$-$3.437x10^4$)	65	$2.031 x10^3$ ($1.409 x10^3$-$3.839 x10^3$)	27	$2.353x10^3$ ($1.918x10^3$-$4.849x10^3$)	19	$1.693x10^3$ ($1.251x10^3$-$2.697x10^3$)	20	p = 0.095
Tie-2	95.02 (70.55-110.34)	65	$1.234x10^5$ ($0.826 x10^5$-$1.368x10^5$)	25	$1.130x10^5$ ($0.781x10^5$-$1.330x10^5$)	19	$5.913x10^5$ ($4.533x10^5$-$8.550x10^5$)	20	**p < 0.001**
PlGF	95.02 (70.55-110.34)	65	100.90 (83.39-109.38)	26	102.57 (78.90-124.83)	19	74.76 (60.39-99.99)	20	p = 0.096
Flt-1	$1.826x10^3$ ($1.459x10^3$-$2.379x10^3$)	65	$1.998 x10^3$ ($1.535 x10^3$-$2.324 x10^3$)	26	$1.826 x10^3$ ($1.663x10^3$-$2.461x10^3$)	19	$1.528x10^3$ ($1.275x10^3$-$2.394x10^3$)	20	p = 0.341

Biomarker levels at baseline were presented with medians and corresponding interquartile ranges (IQR). All values are given in pg/ml. PsA, psoriatic arthritis; TNFi, Tumor Necrosis Factor alpha inhibitor; IL-17Ai, interleukin-17 inhibitor; MTX, methotrexate; VCAM, Vascular Cell Adhesion Molecule; SAA, serum amyloid; ICAM, Intercellular Adhesion Molecule; CRP, C-reactive protein; MIP, macrophage inflammatory protein; IL, interleukin; TNF, Tumor Necrosis Factor; IFN, interferon; TSLP, Thymic Stromal Lymphopoietin; IL-1RA, interleukin 1 receptor antagonist; GM-CSF, Granulocyte-Macrophrage Colony-Stimulating-Factor; TARC, Thymus and activation regulated chemokine; MDC, macrophage-derived chemokine; MCP, monocyte chemoattractant protein; IP-10, IFN-induced protein-10; bFGF, basic Fibroblast Growth Factor; VEGF, Vascular Endothelial Growth Factor; Tie-2, endothelial receptor tyrosine kinase; PlGF, Placental Growth Factor; Fms related Receptor Tyrosine Kinase-1.
