# Supplementary material for: Changes in Inflammatory Cytokines in Responders and Non-Responders to TNFα Inhibitor and IL-17A Inhibitor: A Study Examining Psoriatic Arthritis Patients
Source: Int J Mol Sci. 2024 Mar 5;25(5):3002. doi: 10.3390/ijms25053002 (PMC10932211; doi:10.3390/ijms25053002)
Supplement: Supplementary file 1 [file ijms-25-03002-s001.zip › Table S2.pdf]

Table S2: Log-transformed mean changes in biomarker levels of TNFi initiators stratified by DAPSA50

	**Responders (n=11)**			**Non-responders (n=9)**		
	Mean change	n	p-value	Mean change	n	p-value
VCAM-1	-0.013 (-1.103; 1.075)	11	p = 1.000	0.029 (-1.009; 1.068)	8	p = 0.461
SAA	-0.88 (-4.336; 2.568)	10	p = 0.160	-0.333 (-3.148; 2.483)	7	**p = 0.031**
ICAM-1	-0.072 (-0.946; 0.802)	10	p = 0.557	-0.029 (-1.250; 1.192)	7	p = 1.000
CRP	-1.030 (-4.502; 2.441)	10	**p = 0.049**	-0.013 (-3.888; 3.861)	8	p = 0.844
MIP-3α	-0.323 (-2.191; 1.545)	11	p = 0.240	0.191 (-2.075; 2.458)	7	p = 0.834
IL-27	-0.123 (-1.234; 0.987)	11	p = 0.054	-0.094 (-2.455; 2.267)	8	p = 0.383
IL-23	0.084 (-2.149; 2.316)	8	p = 0.195	0.173 (-3.137; 3.482)	6	p = 0.688
IL-22	-0.094 (-2.499; 2.311)	11	p = 0.520	-0.390 (-3.526; 2.745)	7	p = 0.578
TNFα	0.251 (-2.163; 2.665)	11	p = 0.831	-0.372 (-3.904; 3.160)	9	p = 0.820
IL-8	-0.202 (-1.516; 1.112)	11	p = 0.148	-0.192 (-2.256; 1.873)	9	p = 0.652
IL-6	-0.965 (-4.303; 2.373)	11	**p = 0.032**	-0.308 (-2.380; 1.765)	9	p = 0.098
IL-12p70	0.587 (-2.296; 3.470)	10	**p = 0.049**	-0.185 (-2.270; 1.901)	4	p = 0.875
IL-10	0.381 (-1.740; 2.501)	11	**p = 0.010**	0.376 (-1.720; 2.472)	8	p = 0.055
IFNγ	-0.038 (-2.569; 2.493)	11	p = 0.966	0.518 (-2.209; 3.244)	8	p = 0.148
IL-1RA	0.078 (-2.974; 3.130)	11	p = 0.465	-0.035 (-1.889; 1.819)	9	p = 0.359
IL-17D	0.235 (-1.383; 1.853)	9	p = 0.055	-0.005 (-1.024; 1.013)	8	p = 0.945
IL-17C	-0.225 (-2.143; 1.693)	8	p = 0.547	-0.263 (-2.741; 2.215)	9	p = 0.250
IL-17B	0.080 (-2.168; 2.330)	8	p = 0.547	0.190 (-2.697; 3.077)	7	p = 0.219
IL-7	-0.281 (-2.090; 1.529)	11	p = 0.320	-0.419 (-2.831; 1.992)	8	p = 0.313
IL-5	0.244 (-2.083; 2.571)	6	p = 0.219	0.780 (-1.763; 3.323)	5	p = 0.125
IL-1α	0.160 (-1.879; 2.200)	9	p = 0.129	0.272 (-2.228; 2.773)	7	p = 0.219
IL-17A	0.325 (-3.131; 3.780)	8	p = 0.461	0.640 (-2.284; 3.564)	6	p = 0.297
IL-16	0.031 (-0.785; 0.848)	11	p = 0.638	0.025 (-0.872; 0.921)	8	p = 0.742
IL-15	0.008 (-0.695; 0.710)	11	p = 0.966	0.003 (-1.009; 1.015)	8	p = 0.945
IL-12/IL23p40	0.157 (-1.940; 2.253)	11	p = 0.067	0.223 (-1.780; 2.246)	9	p = 0.496
TARC	-0.629 (-3.446; 2.188)	11	**p = 0.042**	-0.641 (-3.963; 2.680)	9	p = 0.098
MIP-1β	-0.240 (-2.618; 2.138)	11	p = 0.054	-0.230 (-2.103; 1.643)	9	**p = 0.027**
MIP-1α	-0.084 (-1.636; 1.467)	8	p = 0.313	0.411 (-1.920; 2.743)	6	p = 0.438
MDC	0.076 (-1.718; 1.871)	11	p = 0.413	0.082 (-1.919; 2.083)	9	p = 0.910
MCP-4	-0.031 (-1.740; 1.678)	11	p = 0.278	-0.132 (-1.363; 1.099)	9	p = 0.570
MCP-1	-0.124 (-1.671; 1.423)	11	p = 0.067	-0.003 (-1.579; 1.573)	9	p = 0.570
IP-10	-0.120 (-2.165; 1.765)	11	p = 0.148	0.124 (-1.650; 1.897)	9	p = 0.098
Eotaxin-3	0.108 (-1.875; 2.091)	8	p = 0.313	0.195 (-1.435; 1.825)	8	p = 0.383
Eotaxin	-0.045 (-1.575; 1.485)	11	p = 0.638	-0.027 (-1.191; 1.138)	9	p = 0.910
bFGF	-0.028 (-2.891; 2.836)	11	p = 0.520	-0.070 (-2.520; 2.381)	9	p = 0.820
VEGF-D	-0.074 (-0.983; 0.834)	11	**p = 0.042**	-0.105 (-1.139; 0.930)	9	p = 0.055
VEGF-C	-0.014 (-1.305; 1.276)	11	p = 0.898	-0.230 (-2.580; 2.121)	7	p = 0.578
VEGF-A	-0.095 (-1.911; 1.721)	11	p = 0.320	-0.239 (-2.971; 2.492)	9	p = 0.359
Tie-2	0.048 (-1.168; 1.264)	11	p = 0.365	-0.072 (-1.399; 1.254)	8	p = 1.000
PlGF	0.007 (-0.734; 0.747)	11	p = 0.831	-0.016 (-0.838; 0.807)	9	p = 0.820
Flt-1	0.011 (-0.948; 0.971)	11	p = 0.898	-0.095 (-1.124; 0.933)	9	p = 0.250

Mean changes with corresponding 95% confidence intervals stratified by DAPSA50 treatment response. TNFi, Tumour Necrosis Factor alpha inhibitor; DAPSA, Disease Activity in Psoriatic Arthritis; VEGF, Vascular Endothelial Growth Factor; VCAM, Vascular Cell Adhesion Molecule; TNF, Tumour Necrosis Factor; Tie-2, endothelial receptor tyrosine kinase; TARC, Thymus and activation regulated chemokine; SAA, serum amyloid A; PlGF, Placental Growth Factor; MIP, macrophage inflammatory protein; MDC, macrophage-derived chemokine; MCP, monocyte chemoattractant protein; IP-10, IFN-induced protein-10; IL, interleukin; IL-1RA, interleukin 1 receptor antagonist; IFN, interferon; ICAM, Intercellular Adhesion Molecule; Flt-1, Fms related Receptor Tyrosine Kinase-1; CRP, C-reactive protein; bFGF, basic Fibroblast Growth Factor.
