# Supplementary material for: Changes in Inflammatory Cytokines in Responders and Non-Responders to TNFα Inhibitor and IL-17A Inhibitor: A Study Examining Psoriatic Arthritis Patients
Source: Int J Mol Sci. 2024 Mar 5;25(5):3002. doi: 10.3390/ijms25053002 (PMC10932211; doi:10.3390/ijms25053002)
Supplement: Supplementary file 1 [file ijms-25-03002-s001.zip › Table S3.pdf]

Table S3: Log-transformed mean changes in biomarker levels of IL-17Ai initiators stratified by DAPSA50

	**Responders (n=7)**			**Non-responders (n=12)**		
	Mean change	n	p-value	Mean change	n	p-value
VCAM-1	0.109 (-0.651; 0.860)	6	p = 0.313	0.113 (-0.786; 1.012)	12	p = 0.233
SAA	-1.846 (-6.197; 2.506)	6	p = 0.156	-0.547 (-5.339; 4.244)	8	p = 0.547
ICAM-1	0.080 (-0.790; 0.950)	6	p = 0.438	0.079 (-0.647; 0.805)	12	p = 0.064
CRP	-1.592 (-5.488; 2.303)	6	p = 0.156	0.166 (-2.813; 3.145)	11	p = 0.700
MIP-3α	-0.202 (-3.202; 2.797)	7	p = 0.297	0.320 (-1.019; 1.659)	12	**p = 0.016**
IL-27	-0.694 (-4.328; 2.941)	7	**p = 0.031**	-0.251 (-2.770; 2.268)	11	p = 1.000
IL-23	-0.114 (-1.763; 1.534)	5	p = 1.000	0.063 (-2.937; 3.064)	9	p = 0.203
IL-22	0.565 (-3.290; 4.421)	6	p = 0.313	0.006 (-3.022; 3.033)	11	p = 0.577
TNFα	0.232 (-5.316; 5.781)	7	p = 0.375	0.011 (-1.460; 1.482)	12	p = 0.176
IL-8	-0.525 (-2.289; 1.140)	7	p = 0.109	0.219 (-1.757; 2.195)	12	**p = 0.007**
IL-6	-0.888 (-4.241; 2.464)	7	p = 0.109	0.042 (-1.906; 1.989)	12	p = 0.970
IL-10	-0.021 (-2.692; 2.649)	7	p = 0.375	0.131 (-1.313; 1.575)	12	p = 0.110
IFNγ	0.258 (-2.269; 2.785)	7	p = 0.469	-0.107 (-3.447; 3.232	11	p = 0.966
IL-1RA	-0.381 (-2.906; 2.143)	7	p = 0.578	0.047 (-2.272; 2.365)	12	p = 0.733
IL-17D	0.070 (-1.287; 1.428)	4	p = 1.000	0.130 (-1.327; 1.586)	12	p = 0.380
IL-17C	0.081 (-1.845; 2.008)	5	p = 0.813	0.394 (-1.086; 1.874)	10	**p = 0.010**
IL-17B	-0.549 (-2.921; 1.824)	5	p = 0.313	-0.063 (-1.377; 1.251)	11	p = 0.765
IL-7	-0.861 (-3.053; 1.330)	7	p = 0.109	0.244 (-1.714; 2.202)	12	**p = 0.009**
IL-5	0.519 (-2.317; 3.356)	4	p = 0.625	0.424 (-2.127; 2.976)	8	p = 0.250
IL-1α	-0.133 (-2.590; 2.324)	6	**p = 0.031**	0.112 (-1.869; 2.094)	11	p = 0.577
IL-17A	2.490 (-1.864; 6.845)	7	**p = 0.031**	2.476 (-1.459; 6.410)	10	**p = 0.001**
IL-16	-0.076 (-1.061; 0.909)	6	p = 0.313	0.120 (-1.292; 1.533)	12	p = 0.064
IL-15	-0.023 (-0.878; 0.832)	7	p = 0.832	-0.077 (-0.951; 0.796)	12	p = 0.042
IL-12/IL23p40	0.015 (-2.388; 2.417)	7	p = 1.000	0.112 (-2.335; 2.560)	12	p = 0.850
TARC	-0.807 (-3.027; 1.413)	7	p = 0.156	0.392 (-2.029; 2.813)	12	p < 0.001
MIP-1β	-0.253 (-1.527; 1.021)	7	p = 0.078	0.065 (-1.194; 1.324)	12	p = 0.424
MIP-1α	-0.077 (-1.004; 0.851)	6	p = 0.313	-0.066 (-1.869; 1.738)	5	p = 0.813
MDC	-0.078 (-1.317; 1.161)	7	p = 0.230	0.051 (-1.764; 1.866)	12	p = 0.519
MCP-4	-0.348 (-1.819; 1.123)	7	p = 0.219	0.099 (-1.287; 1.486)	12	p = 0.129
MCP-1	-0.151 (-2.453; 2.150)	7	p = 0.375	0.096 (-1.628; 1.820)	12	p = 0.339
IP-10	0.024 (-1.408; 1.457)	7	p = 0.938	0.181 (-1.866; 2.227)	12	p = 0.339
Eotaxin-3	0.080 (-1.906; 2.065)	4	p = 0.875	-0.113 (-2.191; 1.964)	7	p = 0.938
Eotaxin	-0.160 (-1.535; 1.216)	7	p = 0.219	0.074 (-1.231; 1.379)	12	p = 0.733
bFGF	-0.726 (-5.307; 3.855)	7	p = 0.109	0.295 (-2.454; 3.044)	12	p = 0.052
VEGF-D	-0.055 (-1.168; 1.058)	7	p = 0.469	0.66 (-1.203; 1.336)	12	**p = 0.027**
VEGF-C	-0.641 (-2.463; 1.181)	6	p = 0.313	0.192 (-1.277; 1.661)	9	**p = 0.039**
VEGF-A	-0.763 (-2.949; 1.421)	7	p = 0.156	0.160 (-1.766; 2.086)	12	p = 0.151
Tie-2	-0.000 (-1.156; 1.155)	7	p = 1.000	0.029 (-1.821; 1.879)	12	p = 0.204
PlGF	-0.108 (-0.934; 0.718)	7	p = 0.375	0.122 (-1.190; 1.435)	12	p = 0.301
Flt-1	-0.120 (-1.074; 0.834)	8	p = 0.156	-0.091 (-0.998; 0.817)	12	p = 0.339

Mean changes with corresponding 95% confidence intervals stratified by DAPSA50 treatment response. IL-17i, Interleukin-17 inhibitor; DAPSA, Disease Activity in Psoriatic Arthritis; VEGF, Vascular Endothelial Growth Factor; VCAM, Vascular Cell Adhesion Molecule; TNF, Tumour Necrosis Factor; Tie-2, endothelial receptor tyrosine kinase; TARC, Thymus and activation regulated chemokine, SAA, serum amyloid A; PlGF, Placental Growth Factor; MIP, macrophage inflammatory protein; MDC, macrophage-derived chemokine; MCP, monocyte chemoattractant protein; IP-10, IFN-induced protein-10; IL, interleukin; IL-1RA, interleukin 1 receptor antagonist; IFN, interferon; ICAM, Intercellular Adhesion Molecule; Flt-1, Fms related Receptor Tyrosine Kinase-1; CRP, C-reactive protein; bFGF, basic Fibroblast Growth Factor.
