# Supplementary material for: Changes in Inflammatory Cytokines in Responders and Non-Responders to TNFα Inhibitor and IL-17A Inhibitor: A Study Examining Psoriatic Arthritis Patients
Source: Int J Mol Sci. 2024 Mar 5;25(5):3002. doi: 10.3390/ijms25053002 (PMC10932211; doi:10.3390/ijms25053002)
Supplement: Supplementary file 1 [file ijms-25-03002-s001.zip › Table S4.pdf]

Table S4: Log-transformed mean changes in biomarker levels of TNFi initiators stratified by PASI50

	**Responders (n=12)**			**Non-responders (n=6)**		
	Mean change	n	p-value	Mean change	n	p-value
VCAM-1	-0.012 (-0.977; 0.953)	11	p = 0.967	0.013 (-1.096; 1.122)	6	p = 0.563
SAA	-1.183 (-4.401; 2.035)	10	**p = 0.004**	-0.161 (-4.037; 3.715)	5	p = 0.625
ICAM-1	-0.121 (-0.947; 0.704)	10	p = 0.160	-0.023 (-1.252; 1.206)	5	p = 0.813
CRP	-1.058 (-4.722; 2.605)	10	**p = 0.037**	-0.310 (-5.003; 4.384)	6	p = 0.313
MIP-3α	-0.313 (-2.683; 2.057)	11	p = 0.154	0.283 (-0.925; 1.491)	5	p = 0.625
IL-27	-0.187 (-2.126; 1.752)	12	**p = 0.016**	0.056 (-0.990; 1.101)	5	p = 0.813
IL-23	0.158 (-2.935; 3.251)	9	p = 0.359	-0.035 (-2.625; 2.555)	3	p = 0.750
IL-22	-0.622 (-3.523; 2.279)	11	**p = 0.042**	0.621 (-2.044; 3.287)	5	p = 0.125
TNFα	-0.326 (-2.629; 1.977)	12	p = 0.301	-0.112 (-3.041; 2.816)	6	p = 0.844
IL-8	-0.306 (-2.166; 1.555)	12	p = 0.092	0.028 (-0.837; 0.892)	6	p = 1.000
IL-6	-0.961 (-4.182; 2.259)	12	**p = 0.012**	-0.417 (-2.733; 1.899)	6	p = 0.156
IL-13	0.182 (-1.550;1.914)	7	p = 1.000	0.231 (-5.166; 5.627)	3	p = 0.250
IL-12p70	0.272 (-2.219; 2.764)	9	p = 0.129	-0.236 (-2.135; 1.662)	3	p = 1.000
IL-10	0.355 (-2.238; 2.947)	11	**p = 0.010**	0.303 (-1.258; 1.864)	6	p = 0.094
IFNγ	-0.086 (-2.879; 2.798)	11	p = 1.000	0.479 (-2.021; 2.980)	6	p = 0.438
IL-1RA	-0.064 (-1.643; 1.514)	12	p = 0.569	0.050 (-2.956; 3.056)	6	p = 1.000
IL-17D	0.204 (-1.210; 1.619)	10	p = 0.106	0.004 (-0.789; 0.798)	6	p = 1.000
IL-17C	-0.187 (-2.553; 2.178)	9	p = 0.496	-0.141 (-1.388; 1.107)	6	p = 0.563
IL-17B	0.183 (-2.769; 3.135)	8	p = 0.055	0.059 (-2.055; 2.172)	5	p = 1.000
TNFβ	0.070 (-1.625; 1.766)	6	p = 1.000	0.161 (-0.300; 0.623)	3	p = 0.250
IL-7	-0.475 (-2.594; 1.644)	12	p = 0.052	-0.182 (-1.642; 1.279)	5	p = 0.813
IL-5	0.698 (-2.101; 3.498)	6	p = 0.094	0.123 (-0.744; 0.990)	3	p = 0.500
IL-1α	0.274 (-1.643; 2.191)	11	**p = 0.032**	-0.015 (-2.677; 2.648)	3	p = 1.000
IL-17A	0.369 (-3.076; 3.813)	8	p = 0.641	0.772 (-2.789; 4.333)	3	p = 0.438
IL-16	0.007 (-0.757; 0.772)	12	p = 0.733	-0.037 (-1.032; 0.958)	5	p = 0.438
IL-15	0.013 (-0.841; 0.868)	12	p = 0.791	-0.035 (-0.597; -0.528)	5	p = 0.438
IL-12/IL23p40	0.280 (-1.193; 1.753)	12	p = 0.129	-0.041 (-3.359; 3.277)	6	p = 0.563
TARC	-0.923 (-3.465; 1.618)	12	**p = 0.012**	-0.192 (-3.938; 3.554)	6	p = 0.438
MIP-1β	-0.386 (-2.373; 1.600)	12	**p < 0.001**	-0.029 (-1.496; 1.439)	6	p = 0.844
MIP-1α	-0.088 (-1.440; 1.264)	8	p = 0.313	0.593 (-2.119; 3.306)	4	p = 0.625
MDC	0.062 (-1.165; 1.288)	12	p = 0.733	0.132 (-2.302; 2.565)	6	p = 0.438
MCP-4	-0.213 (-1.355; 0.929)	12	p = 0.092	0.096 (-1.653; 1.844)	6	p = 0.844
MCP-1	-0.147 (-1.604; 1.310)	12	p = 0.204	0.073 (-1.395; 1.541)	6	p = 0.844
IP-10	-0.153 (-1.784; 1.478)	12	p = 0.622	-0.035 (-1.860; 1.790)	6	p = 0.844
Eotaxin-3	-0.004 (-1.895; 1.886)	8	p = 0.945	0.238 (-1.149; 1.625)	6	p = 0.219
Eotaxin	-0.152 (-1.568; 1.265)	12	**p = 0.042**	0.169 (-1.068; 1.406)	6	p = 0.156
bFGF	0.026 (-2.603; 2.655)	12	p = 0.569	-0.257 (-3.180; 2.667)	6	p = 0.313
VEGF-D	-0.089 (-0.995; 0.816)	12	**p = 0.021**	-0.091 (-1.250; 1.068)	6	p = 0.313
VEGF-C	-0.123 (-2.128; 1.881)	11	p = 0.831	-0.103 (-1.136; 0.930)	5	p = 0.625
VEGF-A	-0.365 (-2.747; 2.017)	12	**p = 0.042**	0.084 (-2.033; 2.202)	6	p = 1.000
Tie-2	0.009 (-1.349; 1.367)	12	p = 0.266	0.004 (-0.725; 0.733)	5	p = 1.000
PlGF	-0.018 (-0.821; 0.796)	12	p = 0.677	-0.029 (-0.660; 0.607)	6	p = 0.688
Flt-1	-0.063 (-1.128; 1.002)	12	p = 0.380	0.002 (-0.892; 0.895)	6	p = 1.000

Mean changes with corresponding 95% confidence intervals stratified by PASI50 treatment response. TNFi, Tumour Necrosis Factor alpha inhibitor; PASI, Psoriasis Area Severity Index; VEGF, Vascular Endothelial Growth Factor; VCAM, Vascular Cell Adhesion Molecule; TNF, Tumour Necrosis Factor; Tie-2, endothelial receptor tyrosine kinase; TARC, Thymus and activation regulated chemokine; CCL, CC chemokine ligand; SAA, serum amyloid A; PlGF, Placental Growth Factor; MIP, macrophage inflammatory protein; MDC, macrophage-derived chemokine; MCP, monocyte chemoattractant protein; IP-10, IFN-induced protein-10; CXCL, CX chemokine ligand; IL, interleukin; IL-1RA, interleukin 1 receptor antagonist; IFN, interferon; ICAM, Intercellular Adhesion Molecule; Flt-1, Fms related Receptor Tyrosine Kinase-1; CRP, C-reactive protein; bFGF, basic Fibroblast Growth Factor.
