# Supplementary material for: Changes in Inflammatory Cytokines in Responders and Non-Responders to TNFα Inhibitor and IL-17A Inhibitor: A Study Examining Psoriatic Arthritis Patients
Source: Int J Mol Sci. 2024 Mar 5;25(5):3002. doi: 10.3390/ijms25053002 (PMC10932211; doi:10.3390/ijms25053002)
Supplement: Supplementary file 1 [file ijms-25-03002-s001.zip › Table S5.pdf]

Table S5: Log-transformed mean changes in biomarker levels of IL-17Ai initiators stratified by PASI50

	**Responders (n=7)**			**Non-responders (n=4)**		
	Mean change	n	p-value	Mean change	n	p-value
VCAM-1	0.091 (-1.144; 1.326)	6	p = 0.313	0.212 (-0.388; 0.812)	4	p = 0.375
SAA	-0.110 (-4.185; 3.966)	6	p = 0.688	-1.954 (12.045; 8.136)	2	p = 1.000
ICAM-1	0.131 (-0.858; 1.120)	6	p = 0.219	0.095 (-0.408; 0.598)	4	p = 0.125
CRP	0.050 (-3.193; 3.294)	6	p = 0.688	-0.021 (-3.930; 3.889)	4	p = 1.000
MIP-3α	-0.018 (-1.555; 1.519)	7	p = 0.813	0.460 (-1.300; 2.220)	4	p = 0.250
IL-27	-0.621 (-4.075; 0.156)	7	p = 0.156	-0.043 (-0.869; 0.783)	4	p = 0.875
IL-23	0.000 (-3.141; 4.141)	6	p = 1.000	0.092 (-4.747; 4.931)	2	p = 0.500
IL-22	0.517 (-2.091; 3.124)	6	p = 0.438	-0.722 (-5.230; 3.786)	4	p = 0.125
TNFα	0.129 (-5.536; 5.793)	7	p = 0.219	0.237 (-0.527; 1.000)	4	p = 0.125
IL-8	-0.352 (-2.515; 1.811)	7	p = 0.156	0.356 (-0.991; 1.703)	4	p = 0.125
IL-6	-0.055 (-3.305; 3.196)	7	p = 0.813	0.055 (-0.938; 1.049)	4	p = 0.875
IL-10	-0.158 (-2.158; 2.499)	7	p = 0.375	0.059 (-1.190; 1.307)	4	p = 0.875
IFNγ	-0.088 (-3.160; 2.983)	7	p = 0.813	-0.017 (-2.363; 2.330)	3	p = 1.000
IL-1RA	-0.210 (-2.484; 2.064)	7	p = 0.938	0.049 (-1.967; 2.066)	4	p = 0.875
IL-17D	-0.065 (-1.138; 1.008)	6	p = 0.563	0.329 (-0.726; 1.383)	4	p = 0.625
IL-17C	0.307 (-0.952; 1.566)	5	p = 0.125	0.494 (-0.768; 1.755)	4	p = 0.250
IL-17B	-0.812 (-2.642; 1.018)	5	p = 0.125	0.036 (-1.635; 1.707)	4	p = 0.875
IL-7	-0.528 (-3.183; 2.128)	7	p = 0.219	0.113 (-2.245; 2.470)	4	p = 0.625
IL-5	0.586 (-3.150; 4.322)	3	p = 0.750	1.518 (-1.275; 4.312)	2	p = 0.500
IL-1α	-0.096 (-2.563; 2.372)	6	**p = 0.031**	0.262 (-1.047; 1.571)	4	p = 0.625
IL-17A	3.452 (0.095; 6.810)	6	**p = 0.016**	1.742 (-2.930; 6.413)	4	p = 0.375
IL-16	0.011 (-0.726; 0.748)	6	p = 0.688	0.254 (-0.835; 1.344)	4	p = 0.125
IL-15	-0.055 (-1.265; 1.156)	7	p = 0.688	-0.049 (-0.495; 0.396)	4	p = 0.250
IL-12/IL23p40	0.170 (-1.876; 2.216)	7	p = 0.219	0.600 (-2.508; 3.708)	4	p = 1.000
TARC	-0.560 (-3.005; 1.885)	7	p = 0.578	0.360 (-3.368; 4.088)	4	p = 0.125
MIP-1β	-0.285 (-2.582; 2.013)	7	**p = 0.031**	0.250 (-0.489; 0.990)	4	p = 0.250
MIP-1α	-0.173 (-1.068; 0.722)	4	p = 0.250	0.128 (-1.862; 2.118)	2	p = 0.500
MDC	-0.014 (-1.474; 1.447)	7	p = 0.688	0.216 (-2.872; 3.303)	4	p = 1.000
MCP-4	-0.357 (-1.633; 0.919)	7	p = 0.078	0.090 (-2.184; 2.364)	4	p = 0.875
MCP-1	-0.254 (-2.327; 1.820)	7	**p = 0.047**	0.280 (-1.737; 2.298)	4	p = 0.250
IP-10	0.073 (-1,367; 1,514)	7	p = 0.938	0.506 (-0.998; 2.010)	4	p = 0.375
Eotaxin-3	-0.289 (-1.817; 1.239)	4	p = 0.125	-0.391 (-3.794; 3.012)	3	p = 0.500
Eotaxin	-0.243 (-1.749; 1.262)	7	**p = 0.016**	0.259 (-0.985; 1.503)	4	p = 0.625
bFGF	0.009 (-2.949; 2.967)	7	p = 0.938	0.034 (-2.430; 2.499)	4	p = 1.000
VEGF-D	-0.020 (-1.247; 1.207)	7	p = 0.578	0.064 (-1.756; 1.884)	4	p = 0.625
VEGF-C	-0.635 (-2.649; 1.380)	6	p = 0.156	0.198 (-1.644; 2.041)	4	p = 0.375
VEGF-A	-0.618 (-2.499; 1.264)	7	**p = 0.047**	0.452 (-1.884; 2.788)	4	p = 0.250
Tie-2	0.0511 (-1.208; 1.311)	7	p = 0.688	0.042 (-0.637; 0.721)	4	p = 0.625
PlGF	0.112 (-1.010; 1.234)	7	**p = 0.047**	0.248 (-0.826; 1.322)	4	p = 0.625
Flt-1	-0.174 (-1.041; 0.692)	7	p = 0.109	0.692 (-1.041; 0.692)	4	p = 1.000

Mean changes with corresponding 95% confidence intervals stratified by PASI50 treatment response. IL-17Ai, Interleukin 17 inhibitor; PASI, Psoriasis Area Severity Index; VEGF, Vascular Endothelial Growth Factor; VCAM, Vascular Cell Adhesion Molecule; TNF, Tumour Necrosis Factor; Tie-2, endothelial receptor tyrosine kinase; TARC, Thymus and activation regulated chemokine; CCL, CC chemokine ligand; SAA, serum amyloid A; PlGF, Placental Growth Factor; MIP, macrophage inflammatory protein; MDC, macrophage-derived chemokine; MCP, monocyte chemoattractant protein; IP-10, IFN-induced protein-10; CXCL, CX chemokine ligand; IL, interleukin; IL-1RA, interleukin 1 receptor antagonist; IFN, interferon; ICAM, Intercellular Adhesion Molecule; Flt-1, Fms related Receptor Tyrosine Kinase-1; CRP, C-reactive protein; bFGF, basic Fibroblast Growth Factor.
